# Pre-Exposure Prophylaxis to Prevent Hematophagous Bat-Mediated Rabies Outbreaks in Remote Amazon Communities: Lessons from a Pilot for Public Health Policy

**DOI:** 10.3390/tropicalmed9080179

**Published:** 2024-08-14

**Authors:** Felipe Rocha, Alexander Vargas, Elke Maria Nogueira de Abreu, Julio Cesar Augusto Pompei, Marco Antonio Natal Vigilato, Daniel Magalhães Lima, Raphael Schneider Vianna, Ottorino Cosivi, Sergio E. Recuenco, Wagner Augusto Costa, Luciana Hardt, Karin Correa Scheffer Ferreira, Rene dos Santos Cunha Neto, Luciana Botelho Chaves, Andrea de Cassia Rodrigues da Silva, Alberto Lopes Begot, Jorge Alberto Azevedo Andrade, Weber Marcos, Silene Manrique Rocha, Francisco Edilson Ferreira Lima Junior, Marcelo Yoshito Wada

**Affiliations:** 1 Pan American Center for Foot-and-Mouth Disease and Veterinary Public Health–Pan American Health Organization/World Health Organization (PANAFTOSA/VPH-PAHO/WHO), Rio de Janeiro 25045-002, RJ, Brazil; juliopompei@gmail.com (J.C.A.P.); vigilato@paho.org (M.A.N.V.); magalhadan@paho.org (D.M.L.); viannarap@paho.org (R.S.V.); cosivio@paho.org (O.C.); 2 Department of Transmissible Diseases, Secretary of Surveillance in Health and Environment, Ministry of Health, Brazil (SVSA/MS), Brasília 70655-775, DF, Brazil; alexander.vargas@saude.gov.br (A.V.); silene.rocha@saude.gov.br (S.M.R.); francisco.edilson@saude.gov.br (F.E.F.L.J.); marcelo.wada@saude.gov.br (M.Y.W.); 3 Zoonosis State Coordination, Secretary of Health of the State of Pará, Brazil (SES-PA), Belém 66093-677, PA, Brazil; elkeabreu@gmail.com (E.M.N.d.A.); gtzoonosespa@gmail.com (A.L.B.); jorge.azevedo@sespa.pa.gov.br (J.A.A.A.); weberhmarcos@gmail.com (W.M.); 4 Preventive Medicine and Public Health Academic Department, Faculty of Medicine San Fernando, National Universidad Nacional Mayor de San Marcos, Peru (UNMSM), Lima 15081, Peru; sergio.recuencoc@unmsm.edu.pegmail.com; 5 Institute Pasteur, Secretary of Health of the State of Sao Paulo, Brazil (IP/SES-SP), São Paulo 01311-000, SP, Brazil; wcosta@pasteur.saude.sp.gov.br (W.A.C.); lucianahgomes@gmail.com (L.H.); ksferreira@pasteur.saude.sp.gov.br (K.C.S.F.); rene.santos@sp.gov.br (R.d.S.C.N.); lbchaves@pasteur.saude.sp.gov.br (L.B.C.); arsilva@pasteur.saude.sp.gov.br (A.d.C.R.d.S.)

**Keywords:** rabies, public health policy, hematophagous bat, *Desmodus rotundus*, riverine population, pre-exposure prophylaxis

## Abstract

In 2018, an outbreak of human rabies caused by the hematophagous bat *Desmodus rotundus* hit the Brazilian Amazon Basin community of Melgaço, Brazil, resulting in the death of 10 people, 9 of them children. The incidence of rabies has been on the rise among populations in conditions of vulnerability in this ecosystem due to human expansion into sylvatic environments and limited access to public health services. To address this issue, in September 2019, a collaborative effort from national, local, and international institutions promoted and executed a pilot for pre-exposure prophylaxis of a population in high-risk areas for hematophagous bat-mediated rabies. This measure is usually only implemented in response to outbreaks. The pilot was conducted in Portel, in a nearby location to the previous outbreak, with the use of fluvial transportation, and 2987 individuals in 411 dwellings were successfully vaccinated. It established a methodology for pre-exposure prophylaxis for populations in conditions of vulnerability, identifying logistics and costs, as well as characterizing the target riverine population regarding risk factors associated with bites by hematophagous bats. This approach offers a proactive measure to prevent future outbreaks and provides valuable insights into how to address the issue of rabies in remote and difficult-to-reach areas.

## 1. Introduction

**Figure 1:**
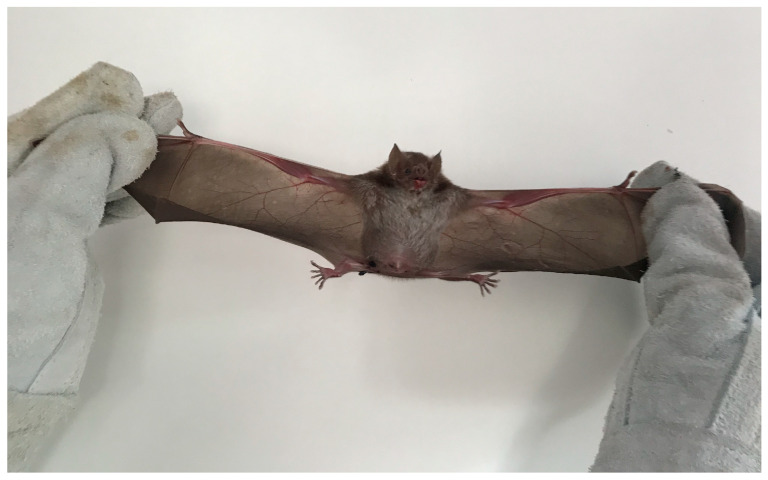
*Desmodus rotundus* bat captured in the proximity of a dwelling in the Pacajá River in Portel, State of Pará, Brazil. Photo by Felipe Rocha. Portel, 2019.

Rabies is an acute zoonotic disease that affects the nervous system and continues to be a major public health concern. While canine rabies is still responsible for 99% of human rabies cases worldwide [1], effective actions and public policies have led to a significant decrease in cases of human rabies mediated by dogs in the Americas [2,3]. As a result of the significant reduction in human rabies mediated by dogs, human rabies through sylvatic cycle has become increasingly apparent, leading to a change in the disease’s epidemiological profile. Rabies mediated by the *Desmodus rotundus* bat—the common vampire bat (Figure 1)—has emerged as a significant public health issue [4]. *D. rotundus* is one of the three species of hematophagous bats found only in the Americas [5] and the most abundant and prolific of these species. When feeding, it bites the mammal’s body and licks the blood that flows from the wound on the skin, thereby transmitting rabies since the disease is mainly spread through saliva [6].

It is, therefore, crucial to continue investing in public health policies and effective actions to combat the spread of rabies by dogs while also addressing the emerging threat of sylvatic-borne rabies. This requires comprehensive measures to protect at risk populations in conditions of vulnerability, living in remote and difficult-to-reach areas where *D. rotundus* bats are prevalent, by raising awareness and investing in proactive measures, such as prophylaxis. Sporadically, outbreaks occur in the Amazon region [7,8,9,10,11], and in Brazil, the last occurred in 2018, leading to the death of 10 people—9 of them were children—who were all riverine residents of the Laguna River area in the municipality of Melgaço, State of Pará [8], one of the locations with the lowest Human Development Index (HDI) in Brazil [12].

**Figure 2:**
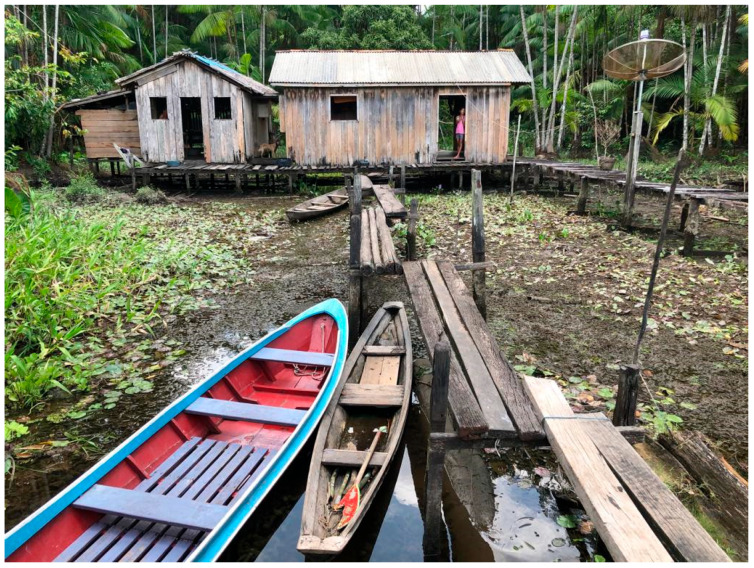
Typical riverine population dwelling distributed along the banks of the rivers of the Amazon Basin. Photo by Júlio Pompei. Portel, 2019.

**Figure 3:**
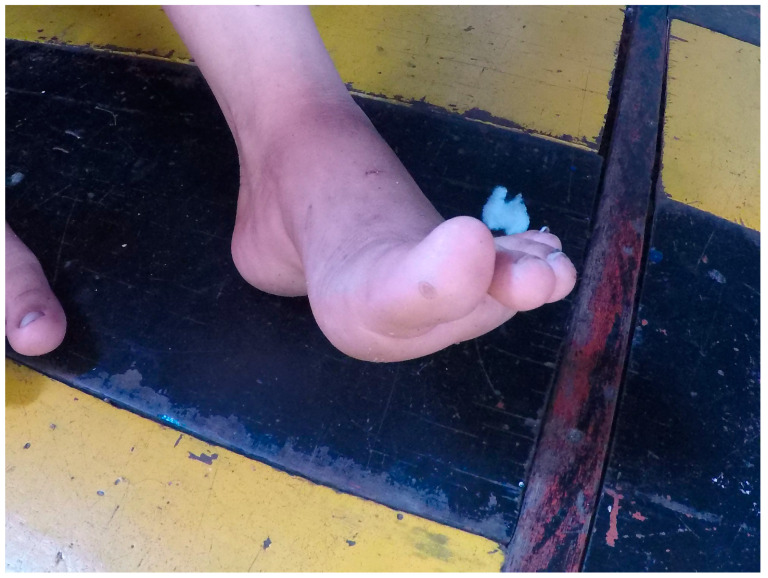
Scar from *Desmodus rotundus* bite on the thumb of an 11-year-old child. Photo by Felipe Rocha. Portel, 2019.

Riverine communities [13] are those who reside on the banks of rivers in remote regions, far from the public health care and basic sanitation services [14,15] offered in urban areas. These communities are surrounded by wild environments and rely on subsistence activities from their surroundings [13]. Due to their proximity and relationship with the environment, riverine communities face various risks and exposures to infectious diseases (Figure 2). As riverine communities are located on the banks of rivers, their dwellings can only be accessed by fluvial transportation, as there are no roads connecting the dwellings across the Amazon jungle. Humans and domestic animals provide a stable feeding source for the common vampire bat [16], leading to an increase in bat–human interactions and the eventual cases of rabies in these environments (Figure 3).

In infected people, when symptoms are presented, considerable portions of the central nervous system are already compromised, with irreversible clinical progressing to death. As rabies has no cure, the prophylaxis of human cases is the best strategy against the disease through vaccination. When applied timely, the vaccine is effective in preventing the development of rabies [17].

Rabies vaccination prevents the disease in humans and can be administered in two ways: post-exposure prophylaxis (PEP) and pre-exposure prophylaxis (PrEP), with intramuscular (IM) or intradermal (ID) protocols. While Brazil only recommends PrEP for certain high-risk professions, the World Health Organization (WHO) recommends PrEP for individuals residing in areas at risk of rabies and with limited access to PEP [17]. The Ministry of Health of Brazil has promoted specific measures to provide PrEP to at-risk populations, although it has not incorporated PrEP as a public health measure because of a lack of information on the logistics and methodology needed [18].

To address this gap, a pilot was designed and conducted in September 2019 by the Ministry of Health of Brazil (MH), the Pará State Secretary of Health (SESPA), and the Municipal Health Secretaries of Breves, Melgaço, and Portel in collaboration with the Pan American Center for Foot-and-Mouth Disease and Veterinary Public Health of PAHO/WHO (PANAFTOSA/VPH-PAHO/WHO) and the Institute Pasteur of São Paulo State Secretary of Health (IP). The main objective of the pilot was to establish a methodology to deploy PrEP as a public health policy and identify the necessary logistics and costs involved for its implementation focused on the riverine populations in high-risk areas for rabies mediated by hematophagous bats and with limited access to PEP. Additionally, the pilot aimed to identify risk factors associated with bites by hematophagous bats and immunological status before PrEP of the riverine population in the area. This initiative was crucial in paving the way for future public health efforts to prevent the occurrence of rabies outbreaks in at-risk populations.

## 2. Materials and Methods

**Figure 4:**
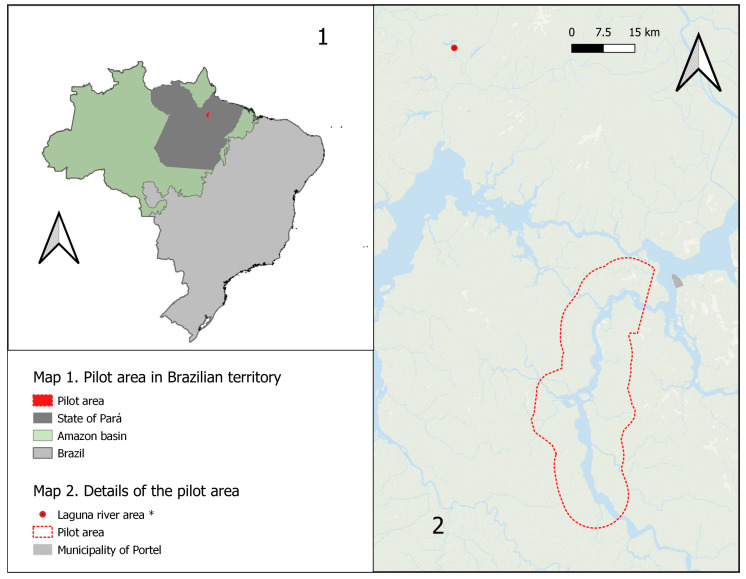
Pre-exposure prophylaxis pilot area in municipality of Portel, PA, Brazil. * Region where the last outbreak of human rabies mediated by hematophagous bats occurred.

The targeted area of the pilot spanned a 60 km stretch along the Pacajá River in Portel [19], PA, Brazil, and extended up to 8 km along tributary rivers, encompassing a total estimated area of 960 km^2^, with a total of 59,441.3 km of rivers to navigate (Figure 4). The selection of this area was based on its proximity and ecological and sociocultural resemblance to the region where the last outbreak of human rabies mediated by hematophagous bats occurred in Melgaço, PA, Brazil, in the Laguna River area [8]. It took place between 9th and 25th September, offering the best conditions to navigate the local rivers.

**Figure 5:**
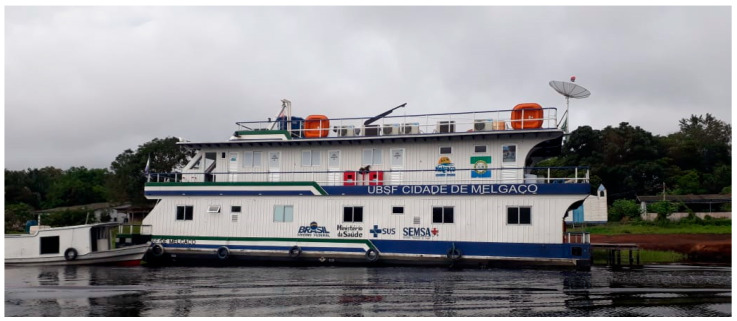
Photo of the Fluvial Basic Unit of Health. Photo by Júlio Pompei. Portel, 2019.

All individuals aged at least 2 years within the designated area received two doses of the VERO rabies vaccine, administered on days zero and seven [17,20,21,22]. Each 0.2 mL ID dose was administered via two applications of 0.1 mL on both forearms. Each vial contained 0.5 mL of vaccine, which was enough to generate two doses of 0.2 mL and half doses of 0.1 mL. The operational base for the pilot was established at a Fluvial Basic Unit of Health (UBSF in Portuguese) (Figure 5) [23]. The infrastructure included six motorboats to facilitate the mobility of teams from the UBSF to dwellings and engaged 41 health professionals.

Blood samples were collected from random individuals, and an epidemiological survey was conducted by healthcare professionals. The survey aimed to gather information on the possible risk factors associated with bites by hematophagous bats, and blood samples were collected as surveillance measure in public health for the titration of neutralizing antibodies against the rabies virus for future follow-up in the incrementation of titers during the following years.

The epidemiological survey gathered information about both individuals and residence characteristics. Individual characteristics included (i) sex, (ii) age (categorized as child—up to 10 years; adolescent—11 to 17; young adult—18 to 30; adult—31 to 60; and elderly—over 60), and (iii) history of bites by hematophagous bats (variable of interest). Residence characteristics comprised (i) presence of domestic animals in the residence; (ii) history of bites by hematophagous bats in animals; (iii) use of lighting at home during the night; (iv) localization of the residence in relation to the Pacajá River; and (v) history of bites by hematophagous bats in the residence (variable of interest). A Multivariate Generalized Linear Model (MGLN) on software R, version 3.5.1 [24] was applied to explore potential correlations among these variables.

For sample size calculation [24,25], the following parameters were applied: expected proportion of 11% of the population with antibodies against rabies [26]; rapid fluorescent foci inhibition test (RFFIT) with 96% sensitivity and 92% specificity [27]; 7% precision (considering the logistic capacity to collect samples); and 95% confidence interval. The result of the sample size was 150 individuals.

Blood samples were collected prior to administering the first vaccine dose and with the written consent of randomly selected individuals. They were labeled and centrifugated, and the resulting supernatant serum was then transferred to microtubes to facilitate their transport from Pará to the laboratory at the IP, São Paulo, to be subjected to the RFFIT test [27,28,29,30].

## 3. Results

The pilot vaccinated 2,987 individuals, of which 1842 individuals received two doses of the vaccine (61.7%), and 879 individuals received one dose (38.3%), distributed in 411 registered dwellings. The registration of 266 individuals was lost. The difference between the total vaccine vials used and the total doses applied and recorded results in 12.5% of lost doses. The estimated total cost of the action, adjusted to present values for January 2024 [31,32], was USD 98,863.03 (Table 1).

**Figure 6:**
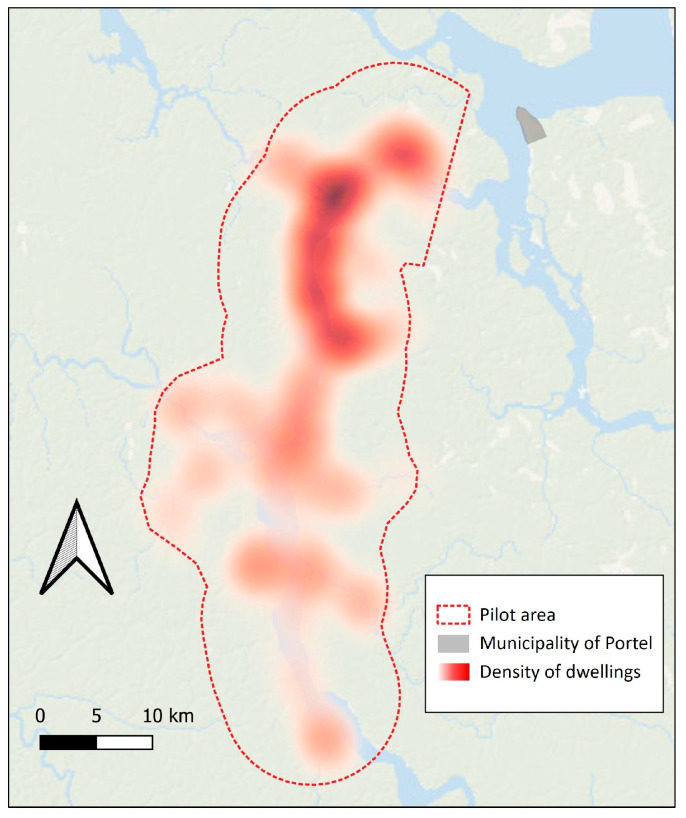
Distribution and heat map of the density of registered dwellings in the pilot area for vaccination.

Table 2 presents demographic characteristics found in the pilot area considering the vaccinated population and the total number of dwellings attended. The coordinates of the dwellings in the pilot area indicate higher density on the banks of the Pacajá River and higher density in the region closer to the urban center of Portel (Figure 6).

A total of 192 individuals were sampled for serological characterization of the population of antibody titers against rabies before vaccination and future serological follow-up; however, only 170 questionnaires were considered due to errors and lack of information in the questionnaires. These 170 individuals were distributed in 105 dwellings and were sampled to characterize the risk of bites by hematophagous bats and for the epidemiological survey. The results of the MGLM for the risk characterization of dwellings and individuals in relation to the occurrence of bites by hematophagous bats can be observed in Table 3. Of the 170 blood samples collected for the titration of neutralizing antibodies, 4 were discarded, and 166 were submitted to the RFFIT test, with an average titer of 0.0291 IU/mL. Of these 166, 2.41% (4 samples, I.C. 0.94–6.03%) presented values equal to or greater than 0.1 IU/mL [26], which are values considered high for individuals who were never vaccinated. Out of the individuals with high antibody titers, only two reported being bitten by hematophagous bats.

## 4. Discussion

The PrEP pilot was a successful collaboration between various public service sectors, reference institutes, and international organizations aiming to promote public health and protect populations in conditions of vulnerability. The pilot provided parameters on how PrEP can be replicated as a public health measure in ecologically and epidemiologically similar areas. To determine which populations were eligible to receive the vaccine, certain criteria should be established, such as assessing the risk of hematophagous bat attacks and limitations on access to public health services. Furthermore, the PrEP pilot is a model of effective cooperation and strategic planning to improve the health outcomes of at-risk populations.

The UBSF that served as the operational base for the pilot was essential in the ecological scenario in which it was carried out. Centralizing the necessary supplies and vaccines supported the vaccinator teams in executing the measures. This structure would not only be valuable for vaccination but would also support the promotion of other health actions in marginalized populations.

Vaccinators with experience in the application of the tuberculosis vaccine [36] were employed to ensure the correct application of ID rabies vaccines. Each dwelling was visited twice, on days 0 and 7, and on some occasions, the number of individuals present in each dwelling varied between the two visits because of labor reasons, for example. Almost 40% of the population received just one dose of the vaccine.

One of the challenges faced was retrieving the entire volume of vaccine from the vials. This was primarily due to factors such as the constant agitation of the small motorboats used to transport the vaccination teams and the design of the vial cap, which resulted in the formation of bubbles. The formation of bubbles and the design of the vial resulted in the loss of doses, meaning that for future PrEP actions under similar conditions, an additional 20% of vials must be considered to ensure that there are enough doses for the entire population.

The environmental conditions also affected the pilot’s records. Although Brazil’s National Immunization Program records show that at least 4829 doses were administered, there were a significant number of individuals who were vaccinated but not registered; records were lost due to adverse working conditions. As a result, the proportion of lost doses may be lower than the 12.5% presented in the results. The wastage seen in the pilot was considerably lower than the 15% of vaccine wastage that is acceptable according to the GAVI Alliance for the COVID-19 vaccine [37], resulting in a vaccine waste cost of USD 7946.00 (the wastage in reality is lower considering the lost records counted as vaccine waste).

The cost of human rabies vaccines accounted for 65.6% of the PrEP pilot’s total cost. An alternative to the ID method would be to adopt the IM protocol, which involves administering two doses of vaccine on days zero and seven [17,19,20,21]; therefore, the IM protocol would require twice as many vaccines, which would represent 78.3% of the total cost of the action and increase the overall cost by 64%. From a financial perspective, the ID protocol has a significant advantage over the IM protocol, as studies have shown that both protocols provide equal immunological protection to vaccinated individuals [17].

When considering operational costs, efforts could be combined to carry out multiple public health measures simultaneously, increasing the efficiency of resource use and promoting health among marginalized populations. The pilot area is also affected by other neglected tropical diseases, such as leishmaniasis, malaria, and yellow fever [38]. The effort to carry out rabies PrEP could be combined with control measures for other diseases, vaccination and surveillance actions, disease treatment distribution, data collection, epidemiological surveys, etc.

There was a higher concentration of dwellings in the region closest to the urban center. The reason for this could be better access to services and infrastructure offered by the city. As one moves away from the urban center toward the rivers, the density of isolated dwellings decreases, and communities and clusters of dwellings become more prevalent.

All mammals, including bats, are susceptible to rabies. Bats contract and die from rabies, and as the symptoms progress, it makes it difficult for a bat to maintain flight and its feeding behavior. To transmit rabies to other mammals, the virus would have to be present in the bat saliva before symptoms appear or at the beginning of the disease. The mechanisms responsible for the maintenance and transmission of rabies between *D. rotundus* bats are grooming, physical disputes, and other social interactions [6]. It is not possible to determine if a bat is positive for rabies unless symptoms are present (suggesting clinical diagnosis) or samples of brain material are taken for rabies diagnosis. This means any bite from *D. rotundus* bats represents a risk for rabies transmission. It is important to note that any wildlife mammal bite event is classified as high risk for rabies by the WHO [16].

The pilot identified that 45.7% of dwellings and 34.4% of individuals in the population had a history of bites by hematophagous bats at least once in their lives. This highlights the risk of rabies that the riverine population in the Amazon Basin faces. It is worth noting that this information relies on the memory of the interviewed individuals.

The presence or absence of domestic animals in the dwellings was not significant to the occurrence of hematophagous bat bites in humans. However, a correlation was found between the occurrence of bites in domestic animals and the location of dwellings with the occurrence of bites in humans. This suggests that the location of dwellings may be a factor that influences the occurrence of hematophagous bat bites in humans. It is worth noting that *D. rotundus* prefers domestic animals over wild animals [39], and this preference may explain the correlation found between the occurrence of bites in domestic animals and the occurrence of bites in humans in nearby dwellings.

The *D. rotundus* is a bat species that relies on a stable feeding source to maintain its territory, as it does not store energy reserves and can die of starvation within two days without feeding [40]. It travels up to 10 km in search of food [41], highlighting the importance of having stable feeding sources within its territory, even if those sources are different mammal or bird species [39]. Dwellings located in areas farther from the main river, such as the Pacajá River, may have easier access as feeding sources for the bats. The affluent rivers of Pacajá enter the interior of the forest, a region of common vampire bat habitats, and as a result, dwellings located in affluent rivers are more exposed to contact with these bats.

No correlation was found between age group or sex and a history of bites. However, it was observed that elderly and adult individuals were more likely to have experienced a bite by a hematophagous bat because of a longer lifetime, raising the chances of being bitten by *D. rotundus*. Eleven individuals claimed to have been bitten by hematophagous bats in recent months, of which four were children, two were adolescents, one was a young adult, and four were adults. Children and adolescents, who were targeted in six of the eleven recent events, had a combined age range of 0–17 years, while young adults and adults had a combined age range of 18–60 years. More than half of the recent attacks had occurred in younger individuals, with a smaller range of ages.

Blood samples revealed the presence of four individuals with rabies antibody titers considered high for those who have never received the vaccine, considering that some individuals who had received the vaccine may not remember doing so. The difficulty in recalling exposure events also could lead to underestimation of bite rates by *D. rotundus* bats, as well as exposure through other sources, such as wild and domestic animals, which also represent a risk for rabies. The highest titer value found was 0.13 IU/mL, which is lower than titers reported in other studies (reaching 2.8 IU/mL) [26].

Overall, there are three recommendations for future PrEP actions to reduce vaccine wastage, decrease the number of individuals receiving only one dose, and prevent the loss of registration: (1) improve communication prior to the action to ensure a higher number of individuals are present at their residences during the vaccinators’ first visits; (2) enhance the quality of training provided to vaccinators, emphasizing the necessity and importance of proper registration; and (3) establish the ID protocol for administering PrEP to populations in conditions of vulnerability in high-risk areas instead of the IM protocol, thereby reducing vaccine costs.

## 5. Conclusions

This pilot project showcases the viability of implementing public policies for PrEP against rabies by administering vaccinations to populations residing in regions susceptible to bites from hematophagous bats. This effort distinguishes itself through its large-scale execution, benefiting thousands of individuals residing in vulnerable conditions, and with that, countries such as Brazil and Perú have been implementing PrEP as a public health policy [42]. It is essential to note that PrEP does not obviate the need for PEP following an exposure incident. However, given the remote nature of the target population, the implementation of PrEP as a public health intervention offers a critical layer of protection. Furthermore, PrEP should be complemented by additional strategies, such as health education and vaccination against other infectious diseases [43]. The targeted population inhabits areas of vulnerability and faces persistent risks of exposure to bites from *Desmodus rotundus* bats, thereby heightening the likelihood of contracting bat-borne rabies. Throughout the pilot project, a commitment to health equity served as a guiding principle embraced by all stakeholders involved.

## Figures and Tables

**Table 1:** Costs of pre-exposure prophylaxis pilot against rabies in a riverine population of the Pacajá River, Portel, Pará.

ITEM	Quantity	Unit Value (BRL)	Present Value * (USD)
1. UBSF [33]—Depreciation	15 days	1,722,060.00 (7300 days of use)	1109.66
2. Small motorboats **—Depreciation	6 units for 15 days	9000.00 (3650 days of use)	69.59
3. Speedboat engines **—Depreciation	6 units for 15 days	13,000.00 (3650 days of use)	100.52
4. Fuel a. Displacement UBSF [33,34] b. Operating UBSF [33,34] c. Motorboats [34]	2880 L 6120 L 1080 L	4.516/L	14,275.36
5. Oil [34]	48 bottles	28.90	435.02
6. Per diem for the professionals **	340 per diem	135.00	14,394.13
7. Inputs (syringes, gloves, cotton, alcohol, artboards, pens, printing, etc.) **			3135.98
8. VERO cells human rabies vaccines [35]	2760 vials	73.45	63,573.13
9. GPS **	6 pieces of equipment	940.50	1769.63
TOTAL (adjusted for January 2024)			98,863.03
Cost per individual (considering 2987 individuals)			33.10
Cost per dose applied (considering 4563 doses)			21.67
Cost by km^2^ covered (considering 960 km^2^)			102.98
Cost by km of rivers navigated (considering 59,441.3 km)			1.66

* Present value in US currency calculated by the multiplication of quantity and unit value (in Brazilian currency) converted by the rate of USD 1.00 equals BRL 5.217 in January 2024 [31,32]. ** Amount paid.

**Table 2:** Demographic characteristics of the pilot population.

Average number of individuals per dwelling (size of family)	7.26 individuals/dwelling
Density (number of individuals per km^2^)	2.83 inhabitants/km^2^
Proportion by sex	Men—50.8% Women—49.2%
Proportion by age	Child—30.50% Adolescent—22.01% Young adult—20.32% Adult—22.31% Elderly—4.85%

**Table 3:** Results of the Multivariate General Linear Model to characterize risk for dwellings and individuals in relation to the occurrence of bites by the *Desmodus rotundus* bat in humans.

Multivariate GLM to Characterize Risk	Correlated Feature	Ratio	Odds Ratio	*p*-Value
Dwellings	Occurrence of bite by *D. rotundus* in domestic animals	66/90	2.85	0.0604
Dwellings	Maintenance of light source at night	70/105	0.79	0.6485
Dwellings	Residence located in affluent rivers	41/105	5.39	0.0008
Individuals	Sex: Male	94/170	1.36	0.372
Individuals	Age category: Adolescent	32/170	0.50	0.282
Individuals	Age category: Young adult	40/170	0.55	0.327
Individuals	Age category: Adult	58/170	1.42	0.518
Individuals	Age category: Elderly	20/170	2.49	0.168

## Data Availability

All data used in the present study are included in the Supplementary Materials, with no mention of a sensitive data origin.

## Supplementary Materials

The following supporting information can be downloaded at: https://www.mdpi.com/article/10.3390/tropicalmed9080179/s1, Table S1. Database of risk factors by dwelling. Table S2. Database of serological results, characteristics and risk factors by individual.
